# Integrative Analysis of Bulk and Single-Cell RNA Sequencing Data Reveals Cell Types Involved in Heart Failure

**DOI:** 10.3389/fbioe.2021.779225

**Published:** 2022-01-05

**Authors:** Xin Shi, Li Zhang, Yi Li, Jieyuan Xue, Feng Liang, Han-wen Ni, Xia Wang, Zhaohua Cai, Ling-hong Shen, Tao Huang, Ben He

**Affiliations:** ^1^ Department of Cardiology, Shanghai Chest Hospital, Shanghai Jiao Tong University, Shanghai, China; ^2^ Key Laboratory of Advanced Theory and Application in Statistics and Data Science, East China Normal University, Ministry of Education, Shanghai, China; ^3^ Bio-Med Big Data Center, Key Laboratory of Computational Biology, Shanghai Institute of Nutrition and Health, Chinese Academy of Sciences, Shanghai, China

**Keywords:** single-cell RNA sequencing, transcriptome, heart failure, dilated cardiomyopathy, ischemic cardiomyopathy

## Abstract

Owing to the high mortality rates of heart failure (HF), a more detailed description of the HF becomes extremely urgent. Since the pathogenesis of HF remain elusive, a thorough identification of the genetic factors will provide novel insights into the molecular basis of this cardiac dysfunction. In our research, we performed publicly available transcriptome profiling datasets, including non-failure (NF), dilated cardiomyopathy (DCM) and ischemic cardiomyopathy (ICM) hearts tissues. Through principal component analysis (PCA), gene differential expression analysis, gene set enrichment analysis (GSEA), and gene Set Variation Analysis (GSVA), we figured out the candidate genes noticeably altered in HF, the specific biomarkers of endothelial cell (EC) and cardiac fibrosis, then validated the differences of the inflammation-related cell adhesion molecules (CAMs), extracellular matrix (ECM) genes, and immune responses. Taken together, our results suggested the EC and fibroblast could be activated in response to HF. DCM and ICM had both commonality and specificity in the pathogenesis of HF. Higher inflammation in ICM might related to autocrine CCL3/CCL4-CCR5 interaction induced chemokine signaling activation. Furthermore, the activities of neutrophil and macrophage were higher in ICM than DCM. These findings identified features of the landscape of previously underestimated cellular, transcriptomic heterogeneity between ICM and DCM.

## Introduction

Heart failure (HF) is a chronic, progressive syndrome with high mortality and mobility, and affects approximately over 37.7 million patients worldwide (Ziaeian and Fonarow, 2016). HF is a serious process of cardiac dysfunction, characterized by impairment of ejection of blood or ventricular filling or both. HF brings a considerable burden to the health-care system, and leads to high rates of hospitalizations, readmissions, and outpatient visits (Bui, Horwich, and Fonarow, 2011; Jones, Roalfe, Adoki, Hobbs, and Taylor, 2019). The rising incidence of HF is associated with multiple factors (Triposkiadis, Xanthopoulos, and Butler, 2019), including age, obesity, hypertension, diabetes mellitus, ischemic heart disease, comorbidities, heredity, and environment, making it difficult to blame it on one specific issue (Oneglia, Nelson, and Merz, 2020; Triposkiadis, Xanthopoulos, Parissis, Butler, and Farmakis, 2020). Since HF is associated with high and unpredictable mortality, there is an emerging interest in potential HF biomarkers, and this exploration benefits the strategies of scientific prevention and advanced therapy.

Complex biological processes are involved in the pathogenesis of HF, and cardiac abnormalities often lead to heart dysfunction. Liu et al.(Liu et al., 2015) collected and analyzed left ventricle issues from six individuals including one ISCH patient, two dilated cardiomyopathy (DCM) patients and three controls as training sets to reveal genetic signatures of HF using RNA-seq and microarray data, which were further validated by a larger cohort with 313 individuals with HF or non-failing (NF). (Sweet et al., 2018) utilized RNA-seq and pathway analysis to reveal the heterogeneous gene signatures and disease-specific mechanisms in 64 explanted human hearts, which consisted of 37 DCM patients, 13 ICM patients, and 14 NF controls. (Vigil-Garcia et al., 2020) applied cardiomyocyte-specific transcriptomic analysis to detect a specific gene set involved in the process of pathological cardiac remodeling related to HF, and they explained the alternations precisely, which occurred during the transition from hypertrophic towards failing cardiomyocytes.

The advances in single-cell RNA sequencing (scRNA-seq) technology offers us an alternative method to characterize cell types involved in HF at the molecular level, which enables its broad application in HF research. (Yamaguchi et al., 2020) manifested that D1R signaling played a pathogenic effect on the process of HF, and explained the association between the activation of D1R and increased risk of patients with HF, using a mouse model of pressure overload-induced HF and single-cell resolution analysis, which aimed to uncover gene expression changes in murine models and human patients at the early and the late stages of HF. (Martini et al., 2019). used single-cell RNA sequencing data to describe the cardiac immune microenvironment in the heart of mouse models with the pressure-overload transverse aortic constriction (TAC) at early and late time points, providing novel diagnostic or therapeutic targets strategies for HF. However, as the sample size of scRNA-seq data is relatively small, and the mechanistic investigation in the variations of some cell types and cell type specific genes involved in HF required the integrative analysis of scRNA-seq and bulk RNA-seq data. In this study, we tried to identify some novel cell types, cell type specific genes and key components in HF by integrating bulk and single-cell RNA sequencing data, and anticipated to reveal cell types involved in DCM and ICM, which will offer a clearer demonstration of the immune inflammation response of HF.

## Materials and Methods

### Data Collection

The single-cell RNA-seq data of two normal left ventricle samples were collected from Gene Expression Omnibus (GEO) with accession number GSE134355 (Han et al., 2020). To identify cell types and key genes related to heart failure, we downloaded the single-cell RNA-seq data of two normal, four dilated cardiomyopathy (DCM), and two ischemic cardiomyopathy (ICM) hearts samples (accession number: GSE121893 (Wang et al., 2020)), one scRNA-seq data of one normal, two DCM and two ICM hearts (accession number: GSE145154 (Rao et al., 2021)) for validation, and bulk RNA-seq data of 14 non-failure (NF), 37 DCM, and 13 ICM samples from GEO database (accession number: GSE116250 (Sweet et al., 2018)). The RNA-seq data of fibroblasts induced by TGFβ1 and control samples, and the microarray-based gene expression data for validation were downloaded from GEO with accession numbers GSE97358 (Schafer et al., 2017) and GSE5406 (Hannenhalli et al., 2006), respectively.

### Cell Clustering Analysis

The unique molecular identifiers (UMIs) count-based scRNA-seq data of the two normal left ventricle samples were used for the cell clustering analysis, which was implemented in R Seurat v3.2.3 package. Cells with less than 500 UMIs were eliminated and features detected in less than 3 cells were filtered. The two hearts were integrated using the anchors by Reciprocal PCA. The expression data was normalized by LogNormalize method with scale factor = 1000,000, and top 2000 highly variable features were selected by FindVariableFeatures with dispersion method. The clusters were found at a resolution of four by FindClusters, and T-distributed Stochastic Neighbor Embedding (t-SNE) was applied to reduce the dimensionality. The cell-type marker genes were detected by FindAllMarkers function at adjusted *p*-value < 0.05, minimal percentage >0.25, and log2 fold change >0.25. All the marker genes of the cell clusters were collected from the earlier study (Han et al., 2020). This analysis was implemented by R Seurat v3.2.3 package (Stuart et al., 2019).

### Principal Component Analysis for the Bulk RNA-Seq Data

The bulk RNA-seq data was downloaded from GEO database (GEO accession number: GSE116250 (Sweet et al., 2018)). The FPKM-based gene expression data were used for PCA analysis. Specifically, gene expressions higher than 1 FPKM in more than five samples were transformed to log2 (FPKM + 1), and the principal components were calculated by R FactoMineR package (Le, Josse, and Husson, 2008) and visualized by R factoextra package.

### Gene Differential Expression Analysis

The pre-normalized microarray data and the RNA-seq data normalized to log2 (FPKM or RPKM +1) were tested by student t test and fold change. The count-based RNA-seq data was processed in R/Bioconductor DESeq2 package (Love, Huber, and Anders, 2014). All *p*-values were adjusted using the Benjamini and Hochberg approach. Genes with an adjusted *p*-value less than 0.05 and a fold change more than two were deemed as differentially expressed genes. Those genes could be ranked by the student *t* statistic to measure the differential expression levels.

### Identification of Cell-types Involved in Heart Failure

The upregulated or downregulated genes in DCM/ICM samples were used for the identification of cell types significantly altered in HF. The gene set overrepresentation enrichment analysis (Fisher’s exact test) was employed to evaluate the significance of the differentially expressed genes (DEGs) against the cell type specific marker genes, which was implemented in R clusterProfiler (Yu, Wang, Han, and He, 2012) package.

### Identification of Endothelial Cell Specific Marker Genes and Cardiac Fibrosis-Related Genes in HF

The gene set enrichment analysis (GSEA) was used to calculate the enrichment degree of those upregulated genes involved in HF or cardiac fibrosis in endothelial cells. Specifically, all the genes were pre-ranked by the t statistic, which represented the differential expression levels. The GSEA analysis was implemented in R clusterProfiler (Yu et al., 2012), and the genes identified as core enrichment in this analysis were considered as key components.

### Gene Set Enrichment Analysis

The gene set overrepresentation enrichment analysis (ORA) was employed to identify the Reactome pathways enriched by previously detected endothelial cell specific marker genes and cardiac fibrosis-related genes in HF. This analysis was implemented in R ReactomePA package and visualized by R clusterProfiler (Yu et al., 2012) package.

### The Cell Activity Estimation

The cell activity was estimated using single-sample Gene Set Variation Analysis (Hanzelmann, Castelo, and Guinney, 2013) (GSVA). Specifically, gene expression profiles and cell type specific marker genes were used as the input for GSVA to estimate the relative activities for each cell type and each sample.

### Statistical Analyses

The two-sample comparison was conducted by student *t* test, and the multiple-sample comparison was implemented by analysis of variance (ANOVA). The *p*-values for multiple-sample comparisons were adjusted to *q*-values by the Benjamini and Hochberg method. Any *p*-values or *q*-values less than 0.05 were considered as statistically significant.

## Results

### Identification and Characterization of Cell Types in Human Left Ventricle

**FIGURE 1:**
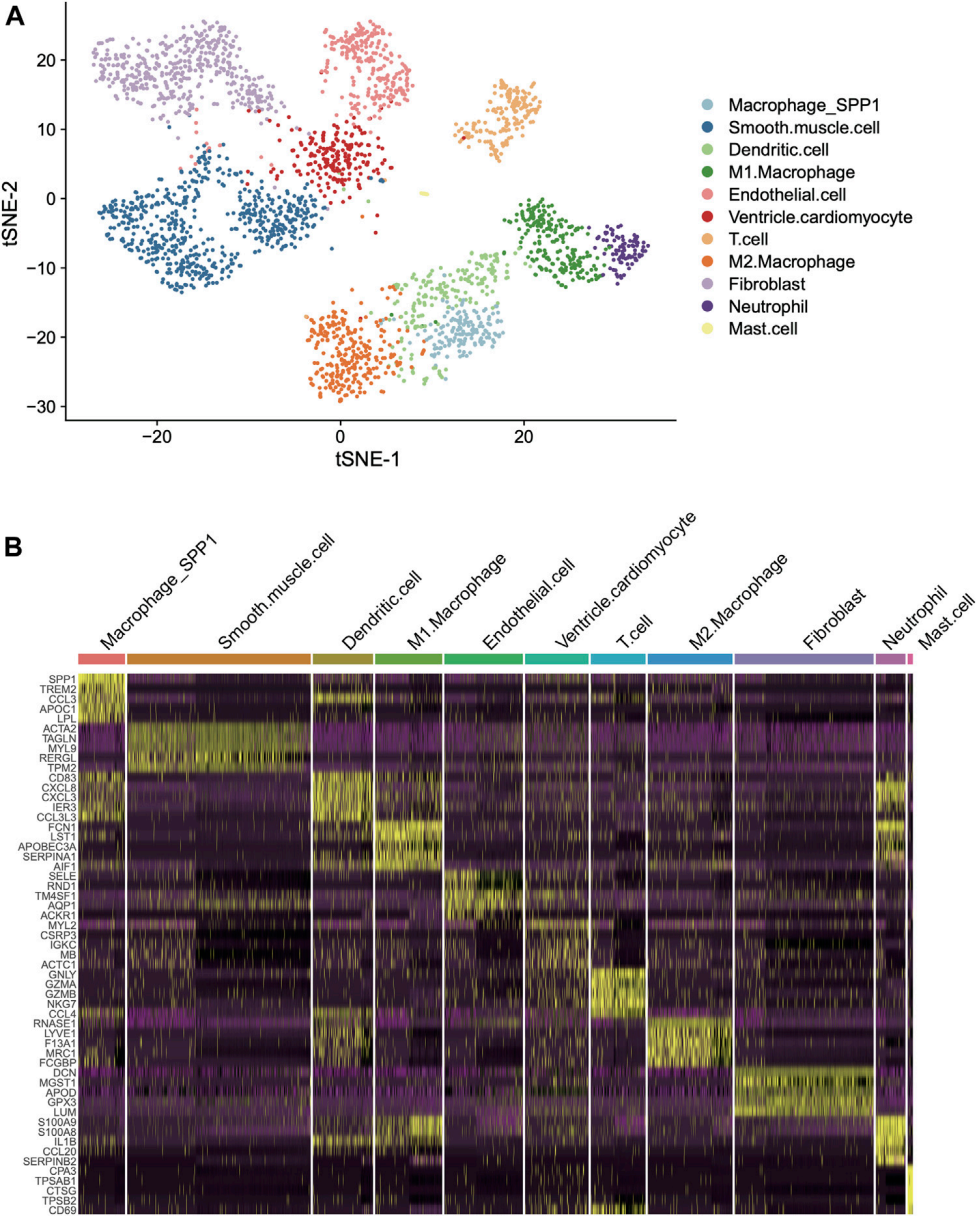
Classification and molecular characterization of the cell types in two human left ventricles. **(A)** The T-distributed Stochastic Neighbor Embedding (t-SNE) analysis for the two left ventricles. Each point represents one cell, and the point colors represent the cell types. **(B)** The expression patterns of the cell type specific maker genes across the cell types in the two hearts (left ventricles).

To identify and characterize the cell types in the human left ventricle (LV), we collected two single-cell RNA sequencing datasets (scRNA-seq) of left ventricle provided by earlier study (Han et al., 2020). Subsequently, we eliminated the cells with low quality and retained 1,324 and 1,480 cells for further analysis (Materials and methods). As shown in Figure 1A, the cells from the two hearts were clustered into 18 clusters by the T-distributed Stochastic Neighbor Embedding (t-SNE) analysis, respectively. Using scHCL method, we successfully annotated 11 cell types for the two hearts (Figure 1A). Notably, the marker genes were specifically expressed in the cell types (Figure 1B). These results indicated that the cell types in the human left ventricle tissues could be identified and well-characterized by the scRNA-seq data.

### The Cell Type Marker Genes Significantly Altered in Heart Failure

**FIGURE 2:**
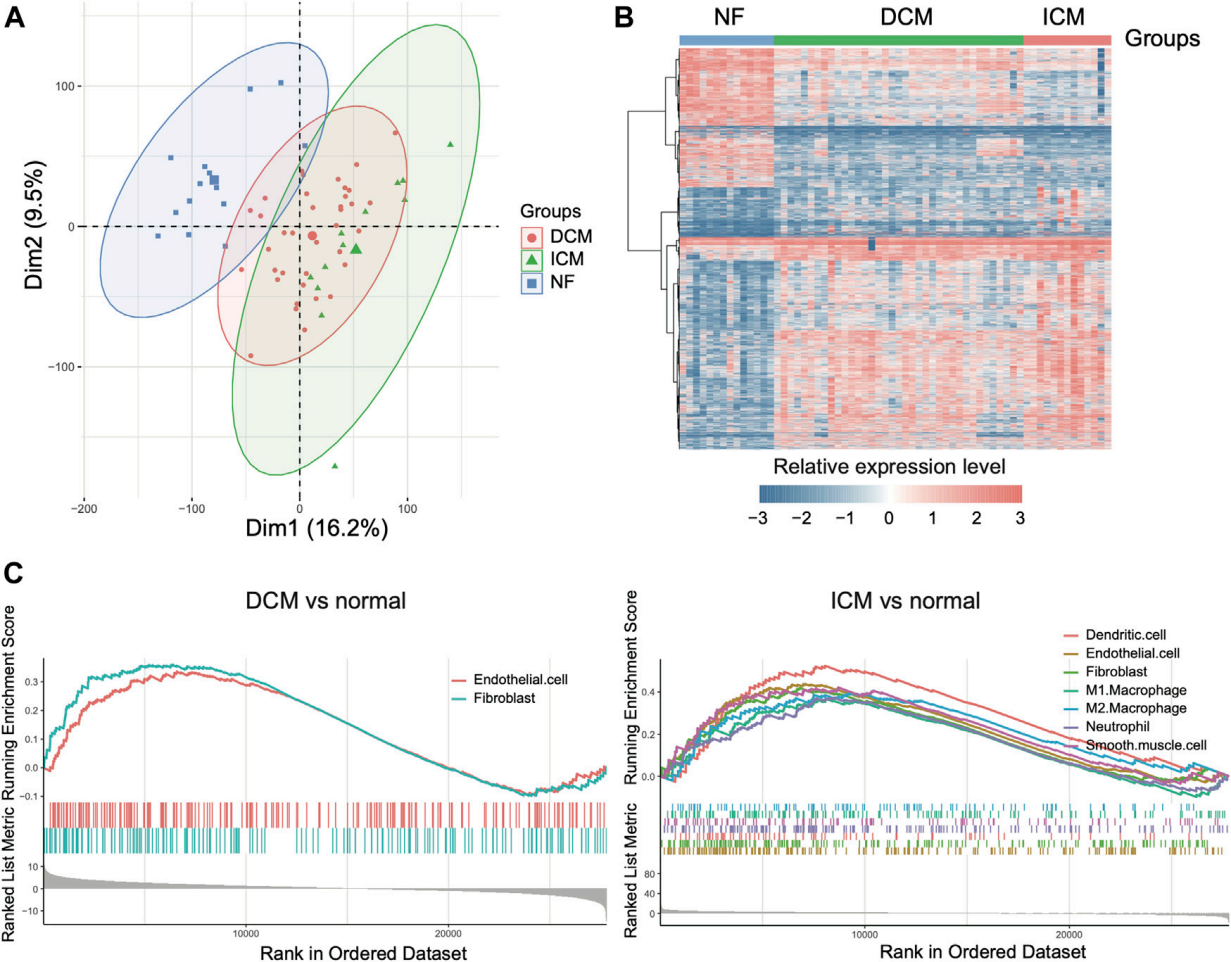
The differentially expressed genes in dilated cardiomyopathy (DCM) and ischemic cardiomyopathy (ICM). **(A)** The scatterplot of principal component analysis for the samples. **(B)** The expression profiles of the differentially expressed genes (DEGs) in DCM and ICM. **(C)** The marker genes of cell types enriched within the upregulated genes of DCM or ICM.

With the cell types and marker genes in the left ventricles, we aimed to identify the cell types altered in the left ventricles of heart failure. We analyzed the gene expression profiles of 14 NF, 37 DCM, and 13 ICM samples from previous study (Sweet et al., 2018). The PCA and differential expression analysis revealed that the samples from the three groups exhibited significantly different expression patterns (Figures 2A,B). Furthermore, we also conducted GSEA on the marker genes of cell types to test whether those marker genes were clustered within the upregulated or downregulated genes of ICM or DCM. Specifically, the marker genes of fibroblast and endothelial cell were significantly enriched within the upregulated genes in both DCM and ICM (Figure 2C, adjusted *p*-value < 0.05), suggesting that the dysfunction of the two cell types might be associated with both DCM and ICM. Moreover, marker genes of dendritic cell, M1/2 macrophage, neutrophil, and smooth muscle cell were more specifically enriched within the upregulated genes in ICM (Figure 2C, adjusted *p*-value < 0.05). These results indicated that DCM and ICM had both similarity and specificity in the pathogenesis of heart failure based on these disease-related cell types.

### Key Regulators in the Endothelial Cells and Fibroblasts of Heart Failure

**FIGURE 3:**
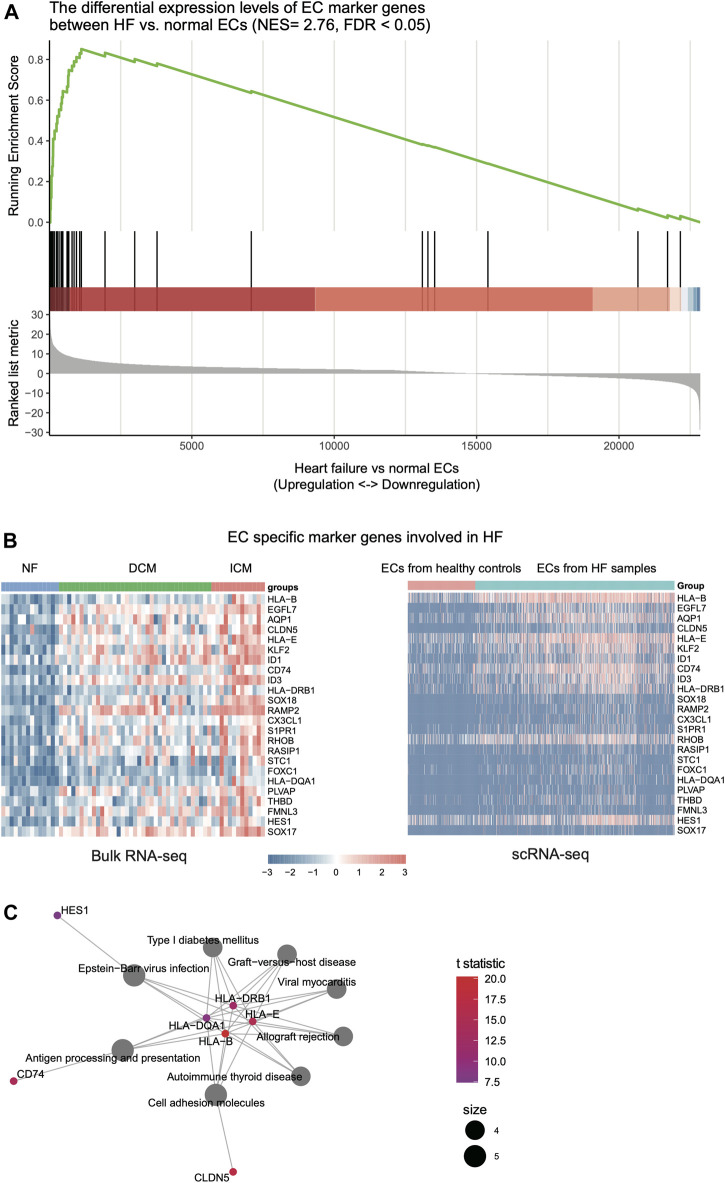
The expression patterns of endothelial cell (EC)-related key regulators involved in HF. **(A)** The genes specifically upregulated in ECs of HF, which are identified by the gene set enrichment analysis (GSEA). **(B)** The expression patterns of genes in bulk RNA-seq and scRNA-seq data of ECs. **(C)** The key regulators in ECs by gene set enrichment analysis (GSEA).

As the endothelial cell and fibroblast could be activated in response to HF (Colombo et al., 2005), we then investigated the key regulators in the ECs and fibroblasts of HF, and collected scRNA-seq data of 1,082 endothelial cells from the left ventricles of NF, DCM, and ICM samples (Wang et al., 2020). The comparison of DCM and ICM samples with NF samples revealed that the endothelial cell specific marker genes were highly enriched in the upregulated genes of HF endothelial cells (Figure 3A, FDR <0.05). Specifically, a total of 24 EC marker genes were found to be upregulated in both HF tissues (bulk RNA-seq) and the endothelial cells of HF samples (scRNA-seq) (Figure 3B, *p*-value < 0.05). The pathway enrichment analysis identified inflammation-related cell adhesion molecules (CAMs) as key regulators, including *CD74, HLA-B, HLA-E, HLA-DRB1, HLA-DQA1, HES1* and *CLDN5*, involved in the pathogenesis of HF (Figure 3C, FDR <0.05).

**FIGURE 4:**
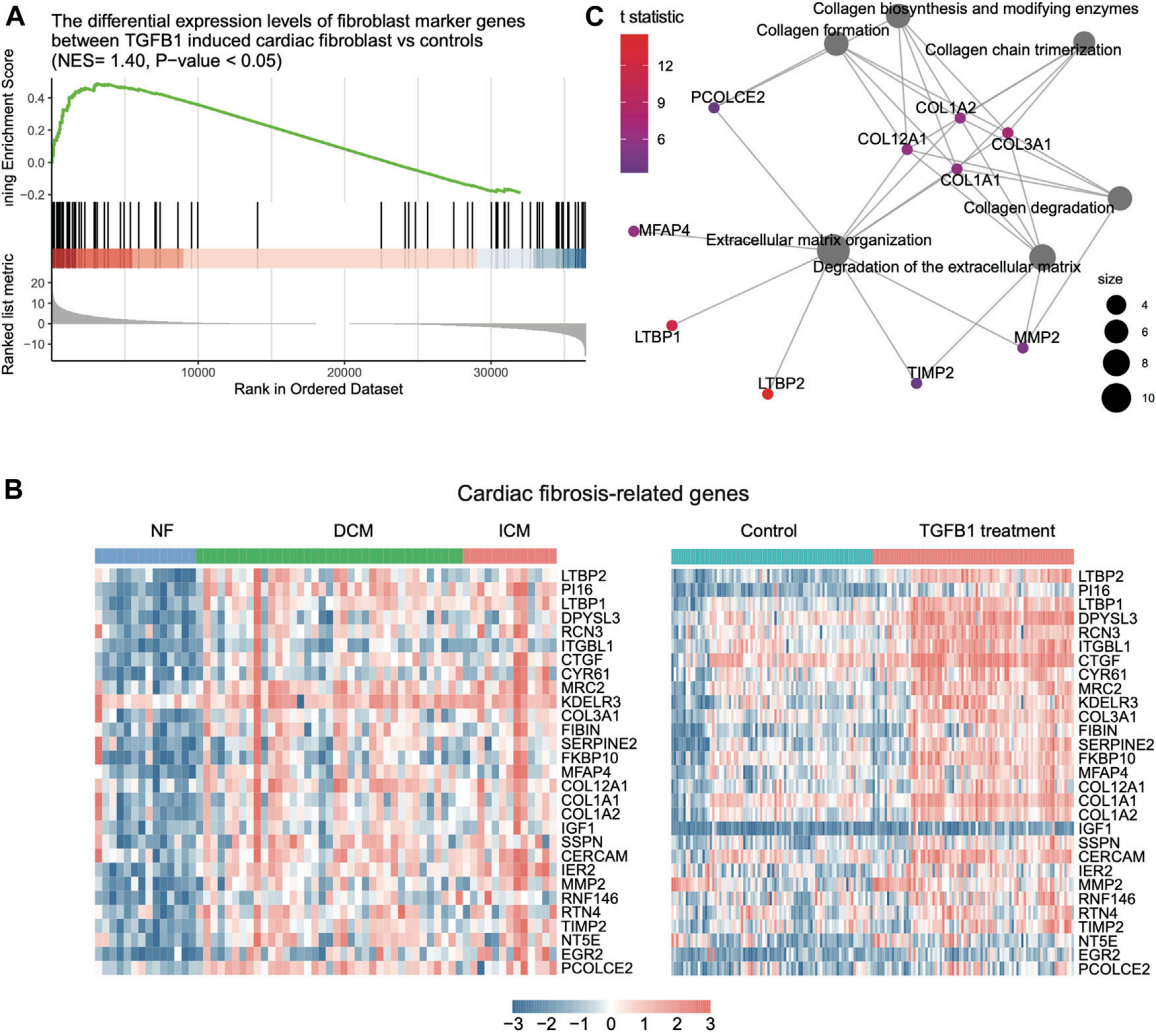
The expression patterns of fibroblast-related key regulators involved in HF. **(A)** The genes specifically upregulated in TGF-beta-induced fibroblast by gene set enrichment analysis (GSEA). **(B)** The expression patterns of cardiac fibrosis-related genes in bulk RNA-seq and scRNA-seq data. **(C)** The key regulators involved in cardiac fibrosis by gene set enrichment analysis (GSEA).

Furthermore, as transforming growth factor β1 (TGFβ1) is the principal pro-fibrotic factor in fibroblast activation (Akhurst & Hata, 2012), (Davis & Molkentin, 2014), which played vital roles in cardiac fibrosis (Ma, Iyer, Jung, Czubryt, & Lindsey, 2017), we examined whether the upregulated fibroblast marker genes in HF were involved in cardiac fibrosis. Consistently, we identified a large proportion of fibroblast marker genes upregulated in TGFβ1 induced cardiac fibroblast by differential expression analysis and GSEA (Figure 4A, FDR <0.05). Among these fibroblast marker genes, 29 were also upregulated in both HF tissues and fibroblast with TGFβ1 treatment (Figure 4B, FDR <0.05). The functional characterization of these genes revealed that *LTBP2*, *LTBP1*, *COL3A1*, *MFAP4*, *COL12A1*, *COL1A1*, *COL1A2*, *MMP2*, *TIMP2*, and *PCOLCE2* were primarily involved in extracellular matrix (ECM) organization and collagen biogenesis/formation/degradation (Figure 4C, FDR <0.05). Collectively, these results indicated that inflammation-related CAMs and ECM proteins such as collagens were specifically secreted by endothelial cell and fibroblast, respectively, and might induce cardiac inflammation and fibrosis during heart failure.

### Chemokine Signaling Activation is Associated with Higher Inflammation in ICM

**FIGURE 5:**
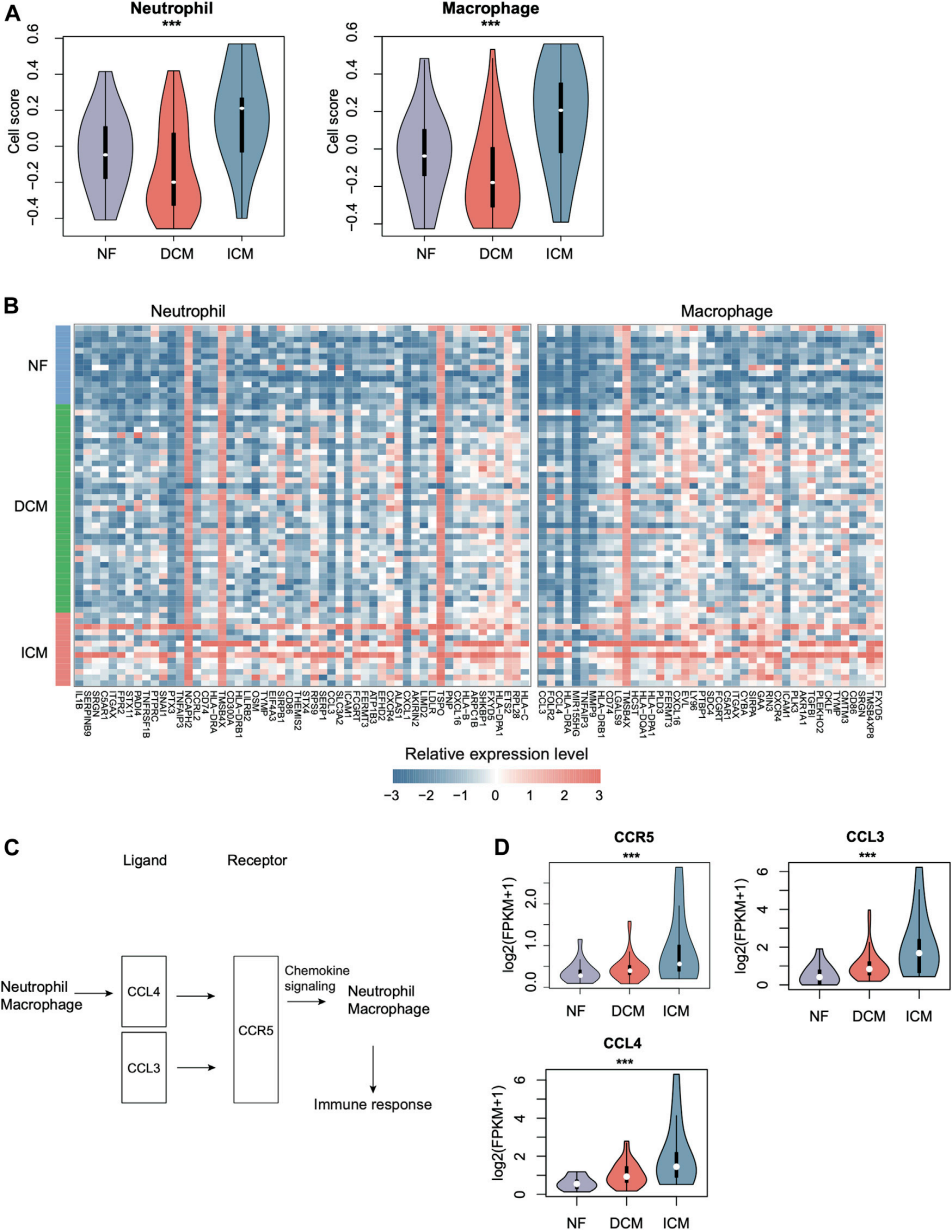
The specific expression patterns of immune cell marker genes in ICM. **(A)** The relative abundances of immune cells including neutrophil and macrophage across the groups. **(B)** The expression patterns of immune cell-specific marker genes in NF, DCM, and ICM samples. **(C)** The autocrine ligand-receptor interactions in neutrophil and macrophage. **(D)** The expression levels of ligands (CCL3/4) and the receptor (CCR5) in NF, DCM, and ICM.

As ICM had more specific immune cell types, such as macrophage and dendritic cell (DC), than DCM, we then estimated the activities of immune cells including macrophage, DC, and neutrophil. Neutrophil and macrophage appeared to have higher activities in ICM than DCM and NF (Figure 5A, *p*-value < 0.05). Consistently, the marker genes of neutrophil and macrophage were also observed to be specifically upregulated in ICM (Figure 5B, *p*-value < 0.05). The cell-cell communication analysis revealed that the autocrine ligand-receptor interaction induced chemokine signaling activation in neutrophil and macrophage might be responsible for the immune response in ICM (Figure 5C). Particularly, the ligands, CCL3, and CCL4, and the receptor CCR5 were specifically upregulated in ICM as compared with DCM and normal controls (Figure 5D). These results indicated that higher inflammation in ICM might be associated with autocrine CCL3/CCL4–CCR5 interaction induced chemokine signaling activation.

### Validation of the Inflammation-Related CAMs, ECM Genes, and Immune Responses in an Independent Dataset

**FIGURE 6:**
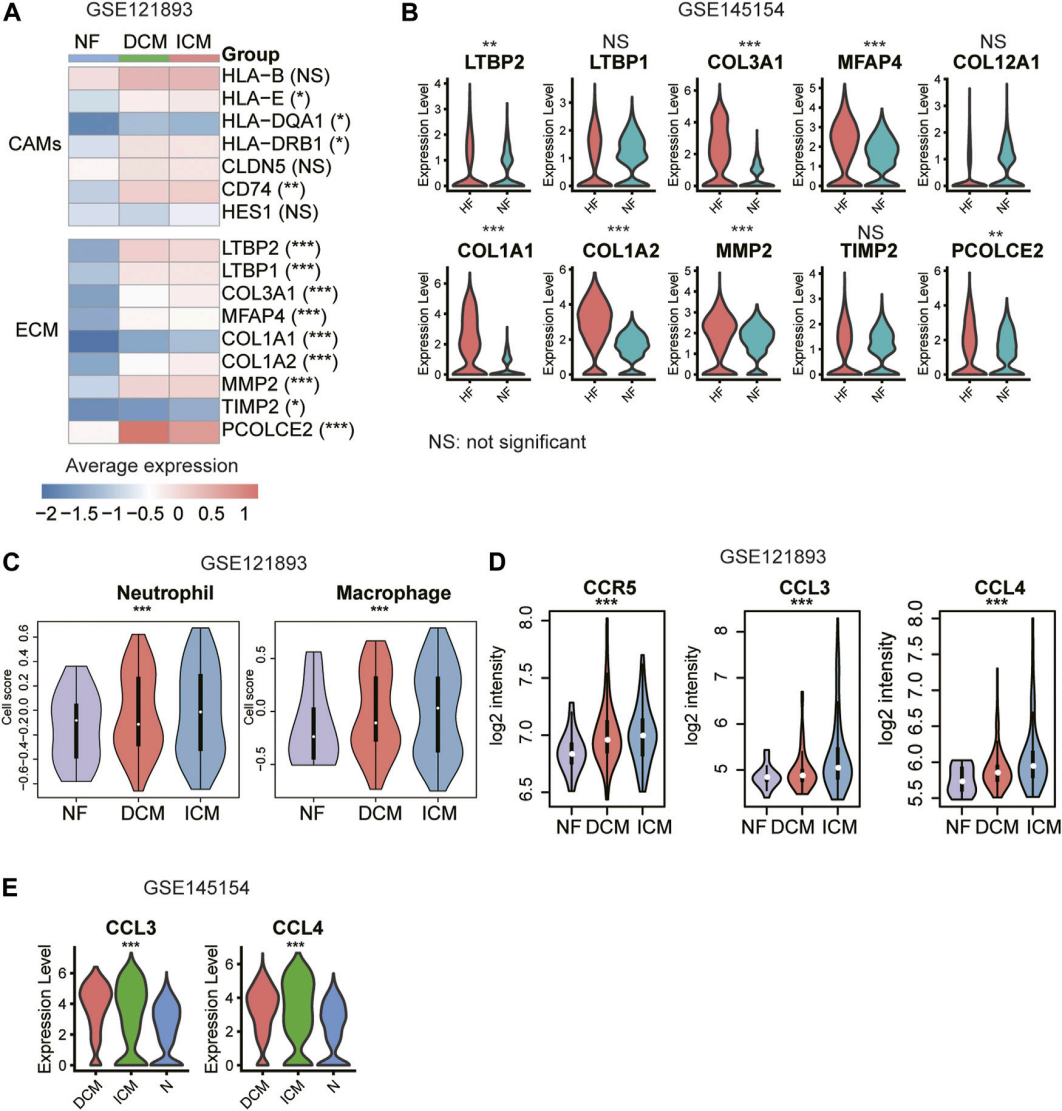
Validation of the cell adhesion molecules (CAMs), extracellular matrix (ECM) genes, and immune responses. **(A)** The upregulation of CAMs and ECM genes in HF samples. **(B)** The differential expression levels of ECM genes between the fibroblasts of NF and HF (scRNA-seq dataset: GSE145154). **(C)** The higher abundance of neutrophil and macrophage in ICM. **(D)** The higher expression levels of CCR5, CCL3, and CCL4 in ICM. **(E)** The differential expression levels of CCL3 and CCL4 between the macrophages of NF, DCM and ICM (scRNA-seq dataset: GSE145154).

We collected an independent gene expression dataset from previous study (Hannenhalli et al., 2006) for validation. The inflammation-related CAMs such as HLA-E, HLA−DQA1, HLA−DRB1, and CD74, and all the ECM genes were upregulated in the HF samples of bulk RNA-seq dataset (GSE121893, Figure 6A, *p*-value < 0.05). Notably, the ECM genes were also upregulated in the fibroblasts of HF from an independent scRNA-seq dataset (Figure 6B). Furthermore, neutrophil and macrophage activities also appeared to be higher in ICM compared with NF and DCM, and the upregulation of autocrine ligand-receptor pairs in ICM, CCL3/CCL4 –CCR5, was also observed in the validation dataset (Figures 6C,D, *p*-value < 0.05). Consistently, the CCL3 and CCL4 were expressed higher in the macrophages of ICM than the DCM and normal hearts (Figure 6E). These results further indicated that inflammation-related CAMs and ECM proteins, which were specifically secreted by endothelial cell and fibroblast, respectively, and chemokine signaling activation in neutrophil and macrophage might induce cardiac inflammation and fibrosis during heart failure.

## Discussion

HF is a major consequence of various cardiovascular diseases with poor prognosis and high mortality (Shantsila, Wrigley, Blann, Gill, & Lip, 2012). In the present study, in order to clarify the cell heterogeneity between ischemic HF and non-ischemic HF, we integrated two scRNA-seq datasets of 1,324 and 1,480 cells from the left ventricles and gene expression profiles of 14 NF, 37 DCM, and 13 ICM samples to identify HF-related cell types and key regulators. Specifically, the marker genes of ECs were significantly upregulated in DCM and ICM proposing that the endothelial dysfunction might be associated with both DCM and ICM. In contrast, DC, M1/2 macrophage, neutrophil, and smooth muscle cell, were specifically upregulated in ICM based on the biomarkers of cell subpopulations. ECs are the most abundant non-myocytes in the healthy heart (Bacmeister et al., 2019). The patterns of endothelial dysfunction in HF patients differed from the etiologies (Oatmen, Cull, and Spinale, 2020). In patients with ischemic HF, endothelial dysfunction is systemic and involves both arteries and veins, conductance vessels and microvascular beds, coronary, pulmonary, and peripheral vessels, however, the patterns of endothelial dysfunction in non-ischemic HF are heterogeneous with fewer features of systemic abnormalities which have a functionally preserved endothelium in peripheral arteries (Berezin, Kremzer, Martovitskaya, Berezina, & Gromenko, 2016).

Fibroblasts as the main effector cells of cardiac fibrosis will be activated after injury associated with HF and participate the process of repair and remodel the infarcted heart (Davis & Molkentin, 2014). Cardiac fibrosis is characterized by an increased amount and a disrupted composition of inflammation-related CAMs and ECM proteins which might be potential targets for heart repair and function (Humeres & Frangogiannis, 2019; Moore-Morris, Guimaraes-Camboa, Yutzey, Puceat, & Evans, 2015). TGF-β1 as a cytokine could induce the transformation of cardiac fibroblasts to myofibroblasts (Akhurst & Hata, 2012). We examined whether the upregulated fibroblast marker genes in HF were involved in cardiac fibrosis through GSEA and differential expression analysis. Among these fibroblast marker genes, 29 were also upregulated in both HF tissues and fibroblast with TGFβ1 treatment. The functional characterization of these genes revealed that they were primarily involved in ECM organization. ECM plays a vital role in cardiac homeostasis, which provides structural support for cardiac cells and maintains integrity and function by transducing important signals among different cells (Frangogiannis, 2019). The transformation of ECM patterns in biochemical in failing hearts hinged on the type of underlying injury (Travers, Kamal, Robbins, Yutzey, & Blaxall, 2016). Collectively, our analysis confirmed that inflammation-related CAMs and ECM proteins such as collagens were specifically secreted by EC and fibroblast, respectively, and might induce cardiac inflammation and fibrosis during the progression of HF.

Previous studies have suggested that inflammation is a key factor of cardiovascular disease, with immune cell types such as macrophages and T lymphocytes mediating essential crosstalk in the progression to HF(Abplanalp et al., 2020). Since we found ICM had more specific immune cell types, such as macrophage and DC, we then focused on the activities of immune cells including macrophage and neutrophil. The cell-cell communication analysis revealed that the autocrine ligand-receptor interaction induced chemokine signaling activation in neutrophil and macrophage might be responsible for the immune response in ICM. During the process of cardiac inflammation, immune cells invade the cardiac tissue and coordinate the responses of damaging. Due to the length limitation of this article, we cannot describe all genes in detail. Taken together, our results suggested that higher inflammation in ICM might be associated with autocrine CCL3/CCL4-CCR5 interaction induced chemokine signaling activation. Furthermore, neutrophil and macrophage also appeared to be higher in ICM compared with DCM.

## Data Availability

The datasets presented in this study can be found in online repositories. The names of the repository/repositories and accession number(s) can be found in the article/Supplementary Material.
